# Optimizing footwear for the diabetic foot: Data-driven custom-made footwear concepts and their effect on pressure relief to prevent diabetic foot ulceration

**DOI:** 10.1371/journal.pone.0224010

**Published:** 2020-04-23

**Authors:** Jennefer B. J. Zwaferink, Wim Custers, Irma Paardekooper, Heleen A. Berendsen, Sicco A. Bus

**Affiliations:** 1 Department of Rehabilitation Medicine, Amsterdam UMC, University of Amsterdam, Amsterdam Movement Sciences, Amsterdam, The Netherlands; 2 Penders Voetzorg, Delft, The Netherlands; 3 Department of Rehabilitation Medicine, Reinier de Graaf Gasthuis, Delft, The Netherlands; University of Illinois at Urbana-Champaign, UNITED STATES

## Abstract

**Aims:**

To assess the effect of data-driven custom-made footwear concepts on plantar pressure relief to prevent diabetic foot ulceration.

**Methods:**

Twenty-four neuropathic diabetic patients at high risk of foot ulceration were measured for in-shoe plantar pressures during walking in four data-driven custom-made footwear conditions, an athletic shoe and an off-the-shelf non-therapeutic shoe. Two evidence-based footwear conditions (Shoe-A; Insole-A) follow a scientific-based design protocol, are handmade, and use in-shoe plantar pressure guided optimization. One evidence-based insole condition (Insole-B) uses a barefoot plantar pressure and 3D foot shape-based computer-assisted design and manufacturing (CADCAM) routine. And one insole condition (Insole-C) uses a barefoot and in-shoe plantar pressure and 3D foot shape-based CADCAM design and optimization routine. Patient satisfaction was scored on walking comfort, shoe fit, weight and appearance.

**Results:**

All data-driven footwear conditions significantly reduced metatarsal head peak pressure compared with the non-therapeutic shoe (17–53% relief). Shoe-A and Insole-A showed the lowest metatarsal head peak pressures (mean 112–155 kPa, 90–98% of cases <200 kPa), significantly lower than for Insole-B and Insole-C (mean 119–199 kPa, 52–100% <200 kPa). Patient satisfaction was not significantly different between footwear concepts.

**Conclusions:**

This study proves the offloading efficacy of a scientific-based, handmade, and in-shoe plantar pressure data-driven approach to custom-made footwear design, and advocates its implementation to optimize diabetic footwear for plantar foot ulcer prevention.

## Introduction

Foot ulceration often occurs as late complication in persons with diabetes mellitus; lifetime incidence has been estimated at 19–34% [1]. The risk of ulcer recurrence after healing is high: 40% in the first year, and 60% after three years [1]. Elevated plantar pressure during walking plays a pivotal role in the development of foot ulcers and their recurrence [2,3]. Therefore, to prevent ulcer recurrence, the International Working Group on the Diabetic Foot 2015 guideline and the more recent Dutch and Australian 2017 guidelines recommend the use of custom-made footwear with a demonstrated peak plantar pressure relieving effect of at least 30% compared with usual care or recently worn therapeutic (custom-made) footwear [4,5,6].

To achieve this target in pressure relief, several data-driven custom-made footwear concepts have been developed. They all use plantar pressure analysis in design and/or evaluation to optimize footwear design. One concept uses in-shoe plantar pressure measurements to guide modifications to custom-made shoes and/or insoles to improve pressure relief. Proof of concept studies have shown that this approach effectively reduces peak pressure at high-pressure regions [7,8], and a randomized controlled trial showed that such pressure improvement significantly reduces the risk of ulcer recurrence when the footwear is adequately worn, in comparison with custom-made footwear that did not undergo such improvement [9]. Information on biomechanically effective shoe and insole design principles obtained from these studies and others [10] has been used to design a scientific-based and in-shoe plantar pressure data-driven shoe and insole concept. Another concept uses 3D foot shape and barefoot plantar pressure data as input to a design algorithm from which through computer-assisted design and manufacturing (CADCAM) a custom-made insole is created [11]. These insoles significantly relieve peak pressure by approximately 30% and a randomized controlled trial showed that they reduce the incidence of plantar metatarsal head (MTH) ulcer recurrence compared to traditional, only shape-based, custom-made insole designs [11,12]. A third concept combines the use of barefoot plantar pressure data to design and in-shoe plantar pressure data to evaluate and, if needed, improve, a CADCAM-based custom-made insole (www.diabetec.de). This insole concept is used in clinical practice through partnerships with orthopedic footwear companies, who send pressure and foot shape data to the insole manufacturing company (Ietec, Künzell, Germany). Proof of principle and clinical efficacy of this insole concept have not yet been reported.

While the superior pressure-relieving capacity and clinical efficacy of two of these data-driven footwear concepts has been demonstrated, a comparison between these data-driven concepts in the same patient has not been made to date. Such a comparison can further improve our understanding of the effect and contribution of data-driven footwear design principles on plantar pressure relief and patient satisfaction while walking, and can give valuable information about the design requirements to build the ´most optimal´ shoe for patients with diabetes at high risk for foot ulceration. Therefore, the aim of this study was to compare these data-driven custom-made footwear concepts for plantar pressure relief and patient satisfaction in persons with diabetes who are at high risk for plantar foot ulceration. Based on the proof of concept studies conducted, we hypothesize that these data-driven footwear concepts will show i) significantly more forefoot plantar pressure relief compared to off-the-shelf non-therapeutic shoes, ii) pressure relief below suggested pressure thresholds for prevention in the majority of cases, iii) more pressure relief in fully custom-made shoes compared to custom-made insoles worn in extra-depth diabetic shoes, and iv) no pressure differences between custom-made insole concepts.

## Materials and methods

### Subjects

Twenty-four persons with diabetes who were at high risk for foot ulceration participated in this cross-sectional study. Participants were recruited under the supervision of the rehabilitation specialist from the outpatient diabetic foot clinic at Reinier de Graaf Gasthuis in Delft, the Netherlands. All participants had loss of protective sensation due to peripheral neuropathy and were classified as International Working Group on the Diabetic Foot (IWGDF) risk category 2 or 3 (i.e. presence of peripheral vascular disease and/or foot deformity, or a history of foot ulceration) [13]. Exclusion criteria were: active ulceration, inability to walk (unaided) repeatedly for at least 20 meters, severe foot deformity (i.e. Charcot foot), amputation other than a single lesser toe, and inability to follow the study instructions. Each participant had been previously prescribed with custom-made footwear and was therefore used to wearing such footwear. An equal distribution in participant number between those who are habitual users of fully custom-made shoes and custom-made insoles worn in extra-depth diabetic shoes was strived for. All participants gave written informed consent prior to the start of the study, which was approved by the medical ethics committee of the Academic Medical Center in Amsterdam.

### Footwear concepts

Three data-driven custom-made footwear concepts were tested: the first concept (A) consists of 2 conditions: a fully custom-made shoe that includes a custom-made insole (Shoe-A) and a custom-made insole (Insole-A) that uses the same design principles as the insole for Shoe-A but is worn in an extra-depth off the shelf diabetic shoe. The other two footwear concepts (B, C) are both custom-made insole concepts. The three insole conditions (Insole-A, B, C) were tested in the same extra-depth shoe that is specifically designed for high-risk diabetic patients. This extra-depth shoe consists of a stiff rocker outsole with a pivot point at 60 percent of shoe length and a rocker angle of 20 degrees, and an upper of stretch material (X-DIAB 14, Podartis, Montebelluna, Italy).

Footwear concept A (Shoe-A and Insole-A) was hand-made by an experienced shoe technician and designed from a data-driven protocol that uses scientific knowledge on effective shoe and insole constructions [7–10,14,15]. The shoe technician (W.C.) had 30 years of experience with providing footwear for the diabetic foot, works in a multidisciplinary diabetic foot team and produces approximately 250 pairs of custom made shoes and insoles for diabetic patients per year. For Shoe-A, the shoe was custom-made and created from a last that was obtained from plaster casting the feet in a semi-weight bearing position. The shoe consisted of a stiff rocker-bottom outsole of Poron with a pivot point located at an average 65% (range across patients 59–73%) of shoe length and a rocker angle of an average 14 degrees (range 10–18 degrees). The custom-made insole for Shoe-A consisted of a 5mm-thick micro-cork base added with a 6mm-thick mid-layer of ethylene vinyl acetate (EVA, shore 35-40A). To guide the placement of a metatarsal bar and medial arch support (EVA, shore 50A) on top of the insole base, a positive plaster cast of the foot was created from static foam box impressions of the foot under semi-weight bearing position and a 6x6mm piece of felt was placed at the corresponding first MTH location on the plaster cast, used as a model of the foot on the insole. Static foot impressions under weight bearing conditions, made with a blueprint pedograph (Bauerfeind, Lopharm, the Netherlands), were used by the shoe technician to identify areas of elevated plantar pressure. At their corresponding regions in the insole, 5mm EVA material was removed and padded with 3mm thick Plastazote®, shore 25A. The insoles were finished with a top cover of 3-mm-thick PPT® and on top of that 3-mm-thick Plastazote®.

For Insole-A, a positive plaster cast of the foot was created from the same static foam box impressions used for Shoe-A. The corresponding MTH region on the plaster cast was marked with lipstick and the cast was applied to a 6mm-thick multiform base, to guide the placement of a metatarsal bar and medial arch support (EVA, shore 50A). The insole was finished with a top layer similar to the insole of Shoe-A.

Both Shoe-A and Insole-A were evaluated using in-shoe plantar pressure analysis (Novel Pedar-X) during overground walking and were modified by the shoe technician if forefoot peak pressure was >200 kPa, following a previously published protocol [7,10].

For the second footwear concept, Insole-B, baseline plantar pressures of the participant were collected during comfortable walking. As per protocol of the insole manufacturer, this was done using an F-scan pressure measurement insole (Tekscan, South Boston, USA) that was inserted in a soft-cover walking shell. A 3D foot scan (CYScan, Choose Your Shoes, Heythuysen, the Netherlands) was made from semi weight-bearing foam box impressions of the foot (the same that were used for concept A). Static weight bearing foot impressions on a blueprint pedograph (Bauerfeind) were used by the shoe technician to draw the contours of a metatarsal pad or bar. The barefoot plantar pressure data, 3D foot scan and the static foot impressions were integrated by another shoe technician in a CADCAM process that drove a milling machine to create an insole with a polyurethane base on top of which a highly elastic foam mid layer was placed. The insoles were hand-finished with a top cover. As part of the design of Insole-B, the insoles were evaluated using in-shoe pressure measurement (F-scan) and modified by the technician until peak pressures at identified high pressure regions were 30% reduced compared with baseline (barefoot) peak pressures.

The third footwear concept, Insole-C, specifically aims to reduce MTH peak pressure at regions with a dynamic barefoot peak pressure >450 kPa [11]. For this concept, dynamic barefoot plantar pressure data of the participant was collected using EMED-X (Novel, Munich, Germany). A 3D foot scan (CYScan) was made of the same static semi weight-bearing foam box impressions of the foot that was used for the other concepts. Additionally, the outline of the inner perimeter of the X-DIAB 14 shoe used was drawn. Pressure, 3D foot shape and outline data were integrated into an automated design algorithm that created a metatarsal bar along high-pressure isobars and removed 3mm of insole material at regions with >1000kPa barefoot pressure [11]. This algorithm drove a CADCAM process that manufactured the insole using a computer numerical-controlled milling machine from a Microcel Puff EVA base (shore 35A). The insole was hand-finished with a laminated 4mm-thick fabric-PPT® top cover [11,12].

In Table 1, a summary is provided of the most important design components of the different footwear concepts tested. As control conditions, an athletic shoe (Asics Gel-Nimbus 17) and an off-the-shelf non-therapeutic shoe without any pressure-relieving design principles (Pulman New Comfort) were additionally tested.

**Table 1:** Summary of design and manufacturing components for the data-driven footwear concepts used in the study.

	Shoe-A	Insole-A	Insole-B	Insole-C
Barefoot pressure data	No	No	Yes	Yes
Foot shape data	3D cast mold	3D scan	3D + 2D scan	3D scan
Design	Scientific algorithm	Scientific algorithm	CAD	Scientific algorithm
	Shoe technician input	Shoe technician input	Shoe technician input	CAD
Manufacturing	Handmade	Handmade	CAM	CAM
Evaluation	In-shoe pressure	In-shoe pressure	In-shoe pressure	No
Modification	Yes, if indicated	Yes, if indicated	Yes, if indicated	No

CAD: Computer-assisted design; CAM: Computer-assisted manufacturing. Shoe technician input: see the text in the methods section for explanation

### Procedures

Participants were examined at baseline, which included the collection of demographic and medical history data (age, sex, type and duration of diabetes, body-weight, height, BMI, history of ulceration and/or amputation), clinical assessment of foot deformity, goniometric assessment of ankle and first metatarsal-phalangeal joint mobility, and screening for diabetic neuropathy. Loss of protective sensation was assessed at the hallux, first and fifth MTH of both feet using a Semmes-Weinstein 5.07 monofilament [13]. Vibration perception threshold (VPT) was assessed at the plantar side of the hallux of each foot using a Biothesiometer (Biomedical Instruments, Newbury, OH, USA); VPT was determined three times and averaged. Loss of protective sensation was defined as the inability to feel the pressure of the monofilament at ≥2 of the 3 sites or a VPT >25 Volts.

After clinical examination, barefoot dynamic plantar pressures were measured in four walking trials for each foot using the EMED-X platform (Novel, Munich, Germany) and a two-step approach to the platform [16]. Other assessments for the purpose of designing one or more of the shoes and insoles (see above) were: dynamic plantar foot pressure assessment using the F-scan system (Tekscan, South Boston, USA), foam box impressions of both feet, 3D scans of these foot impressions, and static pedobarography blueprints on carbon-paper sheets.

In a second session, the plastic shoe mold, that is part of custom-made footwear design for Shoe-A, was fitted to the participant’s foot and, if needed, modified before the definitive shoe was made. Additionally, in-shoe plantar pressures for Insole-B were measured using the F-scan system (Tekscan, South Boston, USA) and, if indicated, the technician modified the insoles to achieve a 30% peak pressure relief compared to baseline plantar pressures measured in session one.

In a third session, approximately 10 weeks after baseline, participants were assessed in all six footwear conditions in a random order for in-shoe plantar pressures during comfortable walking along a 20-meter long walking path. All footwear conditions were tested with the Pedar-X system (Novel, Munich, Germany) and sufficient time was taken in-between measurements. Walking speed was assessed using a stopwatch and standardized between and within footwear conditions (maximum ±5% variation allowed); the target speed was identified while testing the first footwear condition. Several walking trials were used for participants to get accustomed to each footwear condition. A minimum of twelve midgait steps per foot were collected per footwear condition [17]. For Shoe-A and Insole-A, if indicated, the shoe technician modified the footwear and in-shoe pressures were reassessed [7].

Directly after measuring in-shoe pressures in each footwear condition, participants were asked to score their level of satisfaction on four domains by drawing a vertical line on a 10-cm Visual Analogue Scale. The four domains were walking comfort, shoe fit, weight of the shoe, and shoe appearance. A score of 10 denoted the highest possible satisfaction.

Of each insole used in the study, the apex height of the metatarsal bar and its proximal-to-distal distance to the center of the MTH impressions on the insole were determined. Apex height was measured using a digital altimeter. Proximal-to-distal apex distance was measured using a ruler, from the apex to the center of impressions made from lipstick that was applied to the metatarsal head regions on the plaster foot after applying the plaster foot to the insole. These measures were used to assess the influence of metatarsal bar height and location on in-shoe plantar pressure.

### Data analysis

The in-shoe plantar pressure distribution pictures of each footwear condition were masked into ten anatomical regions using Novel Multimask Software (version 24.3.20): medial and lateral hindfoot, medial and lateral midfoot, first MTH (MTH1), second and third MTHs (MTH23), fourth and fifth MTHs (MTH45), hallux, second and third toe (dig2-3), fourth and fifth toe (dig4-5). Individual masks were also created for forefoot regions that showed a measured barefoot peak pressure >450 kPa. Mean peak pressures over all collected steps per condition per participant were calculated for each region and the mean peak pressures per anatomical region were averaged for both feet for further analysis. Mean peak pressures for the medial and lateral midfoot were pooled as well as mean peak pressures for the medial and lateral heel regions. The percentage of the 48 cases (i.e. 24 participants times 2 shoes) showing a mean in-shoe peak pressure below a previously indicated target value of 200 kPa for ulcer prevention [2,7,18] was calculated per region for each footwear concept.

To analyze the effect of metatarsal bar height and location on in-shoe peak pressures measured, the hypotenuse of the apex height and apex proximal-to-distal distance to the center of the MTH impression (MTB_dim_) was calculated for MTH1, MTH 2 and 3 combined, and MTH 4 and 5 combined.

### Statistical analysis

Normality tests were used to examine if the data was normally distributed. To assess differences in regional peak pressure and patient satisfaction scores for normally distributed data, repeated measures analysis of variance with adjustment for multiple pairwise comparisons and a post-hoc (Bonferroni) analysis were conducted. For non-normally distributed data, non-parametric testing using Friedman tests were employed. Metatarsal bar apex height and location were compared between footwear conditions using a one-way analysis of variance and a post hoc (Bonferroni) analysis. The correlation coefficient between MTB_dim_ and percentage of peak pressure reduction compared to the non-therapeutic shoe was assessed for each data-driven footwear concept using a Spearman correlation test. All descriptive and statistical analyses were performed using SPSS for windows (IBM SPSS Statistics version 22, Armonk, NY, USA). All tests were performed with a significance level of alpha 0.05.

## Results

The 24 participants were 16 men and 8 women (3 with type 1 and 21 with type 2 diabetes). Mean (SD) age was 65.8 (10.2) years, diabetes duration 17.3 (11.1) years, body mass index 30.6 (5.7) kg/m^2^ and vibration perception threshold 47.3 (4.4) volts. Exactly half of the participants were habitual wearers of fully custom-made shoes, the other half of custom-made insoles worn in pre-fabricated diabetic shoes. Also exactly half of the participants were classified as IWGDF risk category 2, the other half as risk category 3 (i.e. ulcer history).

### In-shoe peak pressure

Mean peak pressures per anatomical region and region of interest for each footwear concept are shown in Table 2.

**Table 2:** Peak pressures (kPa) per region (of interest) for each footwear condition.

	Shoe-A	Insole-A	Insole-B	Insole-C	Athletic shoe	Non-therapeutic shoe	P-value
*Anatomical foot regions*							
MTH1	132 ± 41***	155 ± 52^§^^¶^	173 ± 76^§^	199 ± 79^§^^%^	227 ± 80***	281 ± 103***	< .001
MTH2-3	141 ± 34^$^^¶^	149 ± 31^¶^	171 ± 42^§^	197 ± 60^§^^%^	198 ± 40^§^^%^^&^	302 ± 75***	< .001
MTH4-5	117 ± 34	112 ± 26^¶^	119 ± 31^¶^	134 ± 42^%^^$^	133 ± 38^§^^%^^&^	161 ± 61***	< .001
Hallux	156 ± 41	167± 44	171 ± 41	175 ± 52	185 ± 56^§^	205 ± 73^§^^¶^	< .001
Midfoot	109 ± 30^¶^	120 ± 24^¶^	121 ± 34^¶^	143 ± 36***	94 ± 19^%^^$^^¶^	88 ± 20^§^^%^^$^^¶^	< .001
Hindfoot	190 ± 51	176 ± 40^$^	211 ± 49^%^^¶^	175 ± 38^$^	198 ± 40^%^^¶^^&^	269 ± 51***	< .001
*Regions with barefoot peak pressure >450kPa (n)*							
MTH1 (n = 25)	140 ± 53***	173 ± 64^§^^¶^	201 ± 96^§^	212 ± 99^§^^%^	257 ± 102***	337 ± 127***	< .001
MTH2 (n = 21)	138 ± 41^§^^¶^	160 ± 39^$^^¶^	191 ± 49^§^^%^	223 ± 69^§^^%^	216 ± 46^§^^%^^$^^&^	350 ± 88***	< .001
MTH3 (n = 11)	159 [119–199]^¶^	154 [122–179]	166 [134–234]	190 [160–243]^§^	199 [193–221]	342 [273–403]	< .001
MTH4 (n = 5)	101 [86–132]	108 [82–116]	107 [90–121]	114 [90–144]	168 [138–197]^%^	208 [164–236]^%^^$^^¶^	.002
MTH5 (n = 14)	100 [88–132]	76 [62–99]	84 [78–98]	71 [50–93]	140 [115–169]***	187 [137–228]***	< .001
Hallux (n = 10)	159 [139–177]	191 [143–203]	186 [146–230]	176 [156–212]	190 [148–224]	228 [174–315]^$^	0.006

data are mean ± SD or median [IQR], with n = 24 for the analysis of anatomical foot regions and n as specified for the analysis of regions with a barefoot peak pressure >450 kPa.

*** significant difference vs. all other concepts (p < .05)

^§^ significant difference vs. Shoe-A (p < .05)

^%^ significant difference vs. Insole-A (p < .05)

^$^ significant difference vs. Insole-B (p < .05)

^¶^ significant difference vs. Insole-C (p < .05)

^&^ significant difference between Athletic shoe and Non-therapeutic shoe (p < .05)

All footwear concepts showed significantly lower MTH peak pressures relative to the non-therapeutic shoe (17–53% reduction, p < .022). Only Shoe-A and Insole-A showed significantly lower MTH peak pressures compared to the athletic shoe (12–42% reduction, p < .031). The lowest peak pressures at MTH1, MTH2-3 and hallux were found in Shoe-A, with MTH1 peak pressure (132 ± 41 kPa) being significantly lower than in any other footwear condition (.001≤ p ≤.021). Apart from MTH1, no other foot regions showed significant differences between Shoe-A and Insole-A. The lowest MTH4-5 peak pressures were found with Insole-A, significantly lower compared to Insole-C (p = .002). Between the three insole conditions, the lowest peak pressures were found in Insole-A, with significantly lower MTH peak pressures compared to Insole-C. Peak pressures at MTH4-5 were also significantly lower with Insole-B compared to Insole-C.

Hallux peak pressures were not significantly different between the data-driven footwear concepts (mean 156–175 kPa, p > 0.111). Midfoot peak pressures were lowest in the non-therapeutic shoe (that had no arch support), significantly lower than in all data-driven footwear concepts (p ≤ 0.013), and highest in Insole-C, significantly higher than in any other footwear condition (P ≤ .001). Hindfoot peak pressures were lowest in Insole-C, and significantly lower compared to Insole-B, athletic and non-therapeutic shoe conditions.

A total of 76 regions with a barefoot peak pressure at the MTH or hallux >450 kPa were identified, in 36 feet of 20 participants. In comparing the six footwear conditions, outcomes for this region of interest analysis were comparable with the outcomes for the anatomical region analysis (Table 2).

The percentages of cases with mean peak pressures <200 kPa are shown per anatomical region in Table 3. The highest percentages for the MTH regions were found with Shoe-A and Insole-A: 90–98%, and the lowest with Insole-C: 52–90%. Percentages at the hallux were generally lower than for the MTHs and comparable between data-driven footwear concepts.

**Table 3:** Percentage of cases (n = 48) with mean peak pressure <200 kPa and number of rounds of modification applied per concept.

	Shoe-A	Insole-A	Insole-B	Insole-C	Athletic shoe	Non-therapeutic shoe
MTH1	94	90	62	52	48	23
MTH2-3	92	96	69	63	56	6
MTH4-5	98	98	100	90	96	75
Hallux	83	73	75	73	60	54
Midfoot	98	98	98	88	100	100
Hindfoot	63	73	40	75	58	13
*Number of participants per number of rounds of modifications*						
0 rounds	15	13	3	-	-	-
1 round	5	6	11	-	-	-
2 rounds	2	2	9	-	-	-
3 rounds	2	3	1	-	-	-
Mean number of rounds	0.6	0.8	1.3	-	-	-

The mean number of rounds of footwear modifications required per patient to achieve the pressure criterion for optimization was 0.6 for Shoe-A, 0.8 for Insole-A, and 1.3 for Insole-B (Table 3). Insole-C was not modified, as per design protocol.

### Patient satisfaction

Scores for patient satisfaction are shown in Table 4. Scores for the footwear concepts varied between 5.6 and 8.3 across domains, with highest scores for walking comfort (7.2) and shoe fit (7.7) found for Insole-B, for shoe weight for Insole-C (8.3), and shoe appearance for Shoe-A (6.9). None of the scores were significantly different between the four data-driven footwear conditions (p ≥ 0.183).

**Table 4:** Patient satisfaction scores.

	Shoe-A	Insole-A	Insole-B	Insole-C	Athletic	Non-therapeutic shoe	P-value
Walking comfort	6.3 ± 2.0	5.8 ± 2.8	7.2 ± 2.0*	6.9 ± 2.7*	6.9 ± 3.0	5.1 ± 2.3	0.016
Shoe fit	7.6 ± 2.1	6.6 ± 3.1	7.7 ± 1.8	6.5 ± 2.7	7.4 ± 3.0	5.6 ± 3.2	0.078
Shoe weight	6.9 ± 2.5*	7.5 ± 2.9	8.0 ± 1.7	8.3 ± 1.4	8.4 ± 2.7	9.0 ± 1.0	0.010
Appearance	6.9 ± 2.7*	5.8 ± 3.2	5.6 ± 3.2	5.8 ± 3.1	5.3 ± 3.5	3.7 ± 2.6	0.015

values are mean ± SD Visual Analogue Scale scores, between 0 and 10 (10 = highest possible satisfaction), (n = 24). For the three insole-conditions the same shoe is evaluated.

* significant difference in comparison with the non-therapeutic shoe (p < .05).

### Metatarsal bar location

Results for the metatarsal bar apex height and location are shown in Table 5. Insole-C had the highest (12 mm) and Shoe-A the lowest (6 mm) mean apex height, with all data-driven concepts showing significantly different heights. Insole-C showed the longest distances between the apex of the metatarsal bar and impression of the MTHs (29–40 mm), Shoe-A and Insole-A the shortest (18–24 mm), and all distances were significantly different across footwear concepts. Spearman correlation coefficients between MTB_dim_ and MTH peak pressure reduction varied between 0.145 and 0.359 and were significant for MTH1 and MTH2-3 (p < .001).

**Table 5:** Height of the metatarsal (MT) bar of the insole and distance between apex of the MT bar and center of metatarsal head (MTH).

	Shoe-A	Insole-A	Insole-B	Insole-C
No. of insoles	40	46	44	46
Height of MT bar (mm)	6 ± 1***	9 ± 1***	11 ± 2***	12 ± 0***
Distance MTH1 (mm)	20 ± 6^$^^¶^	21 ± 7^$^^¶^	33 ± 10^§^^%^	29 ± 8^§^^%^
Distance MTH2-3 (mm)	24 ± 6^$^^¶^	23 ± 7^$^^¶^	35 ± 9***	40 ± 7***
Distance MTH4-5 (mm)	21 ± 7^$^^¶^	18 ± 7^$^^¶^	32 ± 9***	37 ± 6***

data are mean ± SD.

*** significant difference vs. all other concepts (p < .05)

^§^ significant difference vs. Shoe-A (p < .05)

^%^ significant difference vs. Insole-A (p < .05)

^$^ significant difference vs. Insole-B (p < .05)

^¶^ significant difference vs. Insole-C (p < .05)

## Discussion

The results of this study show that data-driven custom-made footwear concepts that use plantar pressure measurement for the design and/or guided modification of the footwear can effectively reduce peak pressures under the forefoot in diabetic patients who are at high risk of foot ulceration. All data-driven footwear concepts showed significantly lower MTH peak pressures relative to the non-therapeutic shoe and most concepts relative to the athletic shoe (hypothesis i, supported). Between the data-driven footwear concepts, quite a large variability in peak pressures was found, with Shoe-A and Insole-A showing significantly lower MTH peak pressures compared to Insole-B and Insole-C. Between 90% and 98% of cases for Shoe-A and Insole-A showed MTH peak pressures below a previously identified target pressure of 200kPa that may protect against plantar foot ulcer recurrence (hypothesis ii, supported) [2,7,18]. These outcomes demonstrate the mechanical efficacy and value of using a systematic, scientific-based, and data-driven approach based on in-shoe plantar pressure measurement for footwear design, evaluation and modification, with the goal to optimize diabetic footwear and patient outcome in those at high-risk for foot ulceration.

In Shoe-A, MTH1 peak pressure was significantly lower than in any other data-driven footwear condition, which may be because Shoe-A is a fully custom-made shoe, allowing more options for customization and pressure relief, such as with the shoe outsole, than custom-made insoles worn in pre-fabricated diabetic shoes (hypothesis iii, partly supported, as for only one foot region). Shoe-A and Insole-A showed significantly lower MTH peak pressures compared to Insole-B and Insole-C. Both Shoe-A and Insole-A were handmade (not through CADCAM), use in-shoe plantar pressure analysis to evaluate and, if needed, improve the footwear, use a clear peak pressure target of 200 kPa as criterion for optimization, and incorporate a 6mm-thick dual-density top cover of the insole, which all distinguish the concept A shoe and insole from the other concepts (Insole-B and Insole-C), and may explain the differences found.

Specifically across the three data-driven custom-made insole concepts, Insole-A showed overall lower peak pressures than Insole-B and Insole-C; mean MTH peak pressures were 6–24% lower for Insole-A (hypothesis iv, rejected). How much this is explained by the insole being either handmade (Insole-A) or CADCAM-based (Insole-B and C) is not known; as mentioned above, this is not the only difference in design between these insole concepts. But the importance of shoe-technician input is suggested from this outcome, and also from the fact that Insole-B, showing the second-best peak pressures, uses input of a shoe technician to integrate data in the CADCAM process, whereas Insole-C is fully automated in CADCAM.

As with Insole-A, Insole-B uses in-shoe pressure-guided footwear modification, but Insole-B required twice as many rounds of modification to achieve the pressure criterion for that footwear concept, which was a 30% peak pressure relief in comparison to baseline peak pressures. This suggests that Insole-A was more appropriately designed before pressure evaluation, probably because of using a detailed algorithm based on knowledge from various studies on footwear design efficacy that specifies the design elements and materials used, their hardness, thickness, and location [7,8,10,14–15,19–21]. One of the differences in design principles that may contribute to this outcome is the use of either metatarsal pads or bars. Insole-B uses a metatarsal pad with high peak pressures at MTH2, MTH3 or MTH4 and a metatarsal bar with high peak pressures at MTH1 and/or MTH5. Insole-A (as does Shoe-A) always uses metatarsal bars to relieve any high pressure at any MTH. In 63% of cases with Shoe-A and 54% of cases with Insole-A, peak pressures after initial design were already <200 kPa, requiring no footwear modification, and the average number of modification rounds required over all 24 subjects was much lower compared to that found previously [8,9]. The result of 90–98% of cases with MTH peak pressures <200kPa after modification was also much better than found previously [8]. This is explained by the improvements made in the initial design of the shoe and insole by changing from a more experience-based to a more scientific-based approach. These outcomes clearly demonstrate the development in footwear design over time and the potential of such a scientific-based and data-driven design and evaluation algorithm for clinical footwear practice for high-risk patients.

Mean peak pressures at the hallux region were between 156 and 175kPa and 73–83% of cases showed a peak pressure <200kPa across data-driven footwear concepts. These outcomes are generally positive, but the lack of significant differences found between concepts indicates that few discriminating design aspects can be found. Generally, fewer options are available to relieve the hallux from high pressure compared to the MTHs, for which a metatarsal pad or bar and an arch support can be effectively used [14,15]. Further research should focus more specifically on methods to effectively relieve hallux peak pressures in high-risk patients, so to further improve outcome, as the hallux is a common location for foot ulcers to develop.

Mean peak pressures in the hindfoot were lowest in Insole-C, and significantly lower than in Insole-B and the athletic and non-therapeutic shoe conditions. This is likely due to the cupping of the heel incorporated in this insole concept, a design principle known to relieve plantar heel pressures [22], and recommended to be incorporated in custom-made insole design. Mean forefoot and midfoot peak pressures found in Insole-C were the highest among the data-driven footwear concepts and were also higher than peak pressures found previously in patients with diabetic neuropathy using this footwear concept [11]. Because Insole-C specifically aims to relieve peak pressures at MTH regions with barefoot peak pressures >450 kPa, an explorative analysis of offloading efficacy in these regions was done. The outcomes of this analysis were comparable with the main findings in showing the highest peak pressures with Insole-C. The reasons for these discrepancies are not clear. Some indication may come from metatarsal bar placement. A metatarsal pad or bar reduces MTH peak pressures by redistributing load from the MTHs to the soft tissues and bony structures proximal to the MTHs, but this is dependent on pad or bar location [14,15,20]. Our analysis showed a more proximally located metatarsal bar in Insole-C and we found this to be weakly but significantly correlated with higher MTH peak pressures. Apex height of the metatarsal bar may be another factor, and was significantly higher for insole-C (mean 12 mm) than for the insole of Shoe-A (6 mm) and insole-A (9 mm). These height differences are explained by the way a positive plaster of the foot is created to use in insole design and by the design rules applied, where apex height can go beyond following the patient’s shape of the foot. How much apex height and location contribute to the difference in pressure relief found, remains to be investigated in more detail.

Perceived usability and satisfaction with prescribed footwear are determinants of footwear use in patients with diabetes [23]. Therefore, patient satisfaction is important in footwear provision for patients who are instructed to always wear their prescribed footwear. Patient satisfaction scores on walking comfort, shoe fit, shoe weight and shoe appearance for the footwear concepts tested were generally moderate to good and not significantly different between concepts. For walking comfort, Insole-B and Insole-C worn in the Podartis X-DIAB 14 diabetic shoe scored similar to an athletic shoe, which is often perceived as comfortable to walk in through its cushioning and easy roll-over. Shoe-A scored highest of all footwear conditions for shoe appearance, which is against experience from clinical practice in which patients often complain about the looks of their custom-made shoes. While the results do not allow drawing strong conclusions due to a lack of significant differences found, the mean score of 6.9 for Shoe-A indicates that aesticallly acceptable custom-made shoes can be provided. The variations found between footwear concepts and across patients should be used to identify factors that may determine perceived satisfaction and as input to future designs of custom-made shoes for patients with diabetes at high-risk for foot ulceration.

The study was limited in that we could not test the most deformed feet, as patients had to be able to fit inside off-the-shelf extra-depth diabetic shoes. Outcomes can therefore not simply be generalized to all high-risk patients who are prescribed with fully custom-made shoes. Another limitation of the study is the choice of control conditions used. The primary aim of this study was to compare different data-driven footwear concepts based on existing proof-of-principle studies that these concepts are better in relieving peak plantar pressure than more traditional-design custom-made footwear. We did not intend to investigate whether data-driven custom-made footwear offloads better than off-the-shelf diabetic footwear with prefabricated insole, but adding the latter condition would have made a useful extra comparison. Additionally, a cost-analysis was not done. The additional use of pressure-measurement and foot scanning equipment and the time effort to train people and to conduct measurements will substantially increase costs for footwear design and evaluation and these may be quite different between footwear concepts. Costs should therefore be considered in the context of pressure relief achieved and cost-effectiveness in preventing foot ulceration.

## Conclusions

This study shows the biomechanical efficacy and value of a systematic, scientific-based and data-driven approach using plantar pressure measurements to custom-made footwear design for patients with diabetic neuropathy at high risk of foot ulceration. The lowest peak pressures were achieved with a footwear concept that is handmade and that uses a detailed scientific-based design algorithm and in-shoe plantar pressure guided footwear modifications to further improve the footwear after delivery, using a clearly defined peak pressure target level. An above 90% success rate at the MTHs in terms of achieving this target pressure that may effectively protect against plantar foot ulcer recurrence if patients wear their shoes, suggests that the knowledge and methods are available to work towards designing the optimal custom-made footwear for the individual patient at high-risk for plantar foot ulceration. We advocate for the implementation of this knowledge and approach in clinical footwear practice.

## Supporting information

S1 File (SAV)
